# DNA methylation and expression profiles of placenta and umbilical cord blood reveal the characteristics of gestational diabetes mellitus patients and offspring

**DOI:** 10.1186/s13148-022-01289-5

**Published:** 2022-05-23

**Authors:** Sha Lu, Jiahao Wang, Nisile Kakongoma, Wen Hua, Jiahui Xu, Yunfei Wang, Shutao He, Hongcang Gu, Jiantao Shi, Wensheng Hu

**Affiliations:** 1 Department of Obstetrics and Gynecology, Hangzhou Women’s Hospital (Hangzhou Maternity and Child Health Care Hospital), Hangzhou, Zhejiang People’s Republic of China; 2 The Affiliated Hangzhou Women’s Hospital of Hangzhou Normal University, Hangzhou, Zhejiang People’s Republic of China; 3 Zhejiang Chinese Medical University, Hangzhou, Zhejiang People’s Republic of China; 4 State Key Laboratory of Molecular Biology, Center for Excellence in Molecular Cell Science, Shanghai Institute of Biochemistry and Cell Biology, Chinese Academy of Sciences, Shanghai, 200031 China; 5 Hangzhou ShengTing Biotech Co. Ltd, Hangzhou, Zhejiang People’s Republic of China; 6 Anhui Province Key Laboratory of Medical Physics and Technology, Institute of Health and Medical Technology, Hefei Institutes of Physical Science, Chinese Academy of Sciences, Hefei, Anhui People’s Republic of China

**Keywords:** Gestational diabetes mellitus, Placenta, Umbilical cord blood, Offspring, DNA methylation, Transcriptome

## Abstract

**Background:**

Gestational diabetes mellitus (GDM) is a common pregnancy-specific disease and is growing at an alarming rate worldwide, which can negatively affect the health of pregnant women and fetuses. However, most studies are limited to one tissue, placenta or umbilical cord blood, usually with one omics assay. It is thus difficult to systematically reveal the molecular mechanism of GDM and the key influencing factors on pregnant women and offspring.

**Results:**

We recruited a group of 21 pregnant women with GDM and 20 controls without GDM. For each pregnant woman, reduced representation bisulfite sequencing and RNA-seq were performed using the placenta and paired neonatal umbilical cord blood specimens. Differentially methylated regions (DMRs) and differentially expressed genes (DEGs) were identified with body mass index as a covariate. Through the comparison of GDM and control samples, 2779 and 141 DMRs, 1442 and 488 DEGs were identified from placenta and umbilical cord blood, respectively. Functional enrichment analysis showed that the placenta methylation and expression profiles of GDM women mirrored the molecular characteristics of “type II diabetes” and “insulin resistance.” Methylation-altered genes in umbilical cord blood were associated with pathways “type II diabetes” and “cholesterol metabolism.” Remarkably, both DMRs and DEGs illustrated significant overlaps among placenta and umbilical cord blood samples. The overlapping DMRs were associated with “cholesterol metabolism.” The top-ranking pathways enriched in the shared DEGs include “growth hormone synthesis, secretion and action” and “type II diabetes mellitus.”

**Conclusions:**

Our research demonstrated the epigenetic and transcriptomic alternations of GDM women and offspring. Our findings emphasized the importance of epigenetic modifications in the communication between pregnant women with GDM and offspring, and provided a reference for the prevention, control, treatment, and intervention of perinatal deleterious events of GDM and neonatal complications.

**Supplementary Information:**

The online version contains supplementary material available at 10.1186/s13148-022-01289-5.

## Introduction

Gestational diabetes mellitus (GDM) is a common pregnancy-specific disease, characterized by glucose intolerance. GDM usually occurs in the middle or late pregnancy [1–3]. Increased insulin resistance and impaired insulin secretion are the main causes of GDM [4]. Due to different lifestyles, ethnicities, diagnostic methods, the median (interquartile range) prevalence (%) of GDM varies significantly from the lowest in Europe (6.1, 1.8–31) to the highest in the Middle East and North Africa (15.2, 8.8–20) during 2005–2018 [5]. Independent studies show that factors affecting the occurrence of GDM include maternal weight, gestational weight gain, diet and exercise, ethnicity, maternal age, and family history of diabetes [6–11]. Women with GDM are prone to pregnancy-related complications or perinatal adverse events, such as hypertension, preeclampsia, preterm birth, shoulder dystocia, perinatal morbidity, and death [12]. Fetuses exposed to GDM have an increased risk of macrosomia, fetal distress, congenital malformations, and jaundice [13]. Meanwhile, the offspring of GDM women has an increased risk of metabolic diseases and cardiovascular diseases, such as obesity and type II diabetes. GDM is also related to immune function loss and cognitive impairment in the offspring [14]. The molecular mechanism of insulin resistance and insulin secretion defect in pregnant women is still under intensive investigation.

From a fertilized egg to the complete fetus, human genome remains relatively stable while the epigenome undergoes regulated reprogramming including dramatic DNA methylation changes and systemic histone modification alternations [15, 16]. Moreover, the epigenome of fetus is sensitive to the pregnant women’s intrauterine environment, especially high-glucose levels, which eventually leads to phenotypic changes [17]. How the exposure of fetal to maternal GDM contributes to increasing offspring risk remains to be explored. Results from animal studies suggest that the exposure to intrauterine high-glucose environment may cause disorders of hypothalamic neuropeptide neurons, impair blood glucose homeostasis and lead to abnormal renal development and islet β-cell dysfunction [18, 19]. Epidemiological studies have also confirmed that exposure of intrauterine high-glucose environment may cause the transfer of lipid matrix molecules to the fetus, resulting in disorders associated with adipocyte dysfunction and fatty acid accumulation [20]. Using bisulfite pyrosequencing assays, Houde and colleagues identified three candidate genes, *LRP1B*, *BRD2* and *CACNA1D*, whose DNA methylation alterations in placenta and umbilical cord blood were related to energy metabolism and maternal glucose levels [21]. Through methylated DNA immunoprecipitation (MeDIP) assay and next-generation sequencing (NGS), Rong and colleagues revealed genome-wide changes in the placenta of GDM patients and transcriptional variations of four GDM-related genes [22]. However, the genes and pathways involved in GDM have not been systematically characterized and thus remain elusive. Therefore, genome-scale DNA methylation analysis and transcriptome studying of the placenta and paired umbilical cord blood from women with or without GDM can potentially uncover the underlying molecular mechanism.

Reduced representation bisulfite sequencing (RRBS) is an accurate and cost-effective method that can provide genome-wide mapping 5-methylcytosines, particularly enriching for coverage in promoter and enhancer regions that are crucial for transcriptional regulation [23, 24]. RNA-seq allows to profile thousands of gene expressions precisely and simultaneously [25]. Here, we employed the RRBS and RNA-seq methods to systematically analyze the genome-wide methylation and expression profiles of placenta and paired umbilical cord blood from a group of patients with GDM and control pregnant women without GDM. The aim of this study is to reveal critical epigenetic changes associated with GDM in pregnant women and to identify differentially expressed genes (DEGs) which may potentially contribute to GDM.

## Results

### Clinical characteristics of pregnant women and newborns

Forty-one pregnant women were recruited into this study, including 21 pregnant women with GDM and 20 without GDM as control. Those pregnant women delivered 41 babies, including 4 babies with macrosomia, 2 of which were from the GDM group. Anthropometric and metabolic parameters of pregnant women and newborns are shown in Table 1. We observed that both the maternal weight and body mass index (BMI) were significantly higher in the GDM group compared with those in the control group (Wilcoxon rank-sum test, *P* value < 0.05), while other characteristics, such as maternal age, gestational weight gain, maternal height, gestational weeks, and fetal birth weight, did not show significant differences between two groups. Given the significant differences of maternal BMI between GDM and control groups, BMI will be used as a covariate in the subsequent search for differentially methylated regions (DMRs) and DEGs.

**Table 1:** Anthropometric and metabolic parameters of pregnant women and newborns

Parameters	GDM (*n* = 21)	Control (*n* = 20)	*P* value	Difference
Maternal age (years)	30.95 ± 3.8	30.05 ± 3.17	0.64	n.s
Maternal birth weight (kg)	73.56 ± 10.71	66.86 ± 6.08	0.029	*
Gestational weight gain (kg)	12.22 ± 3.01	14.25 ± 3.85	0.073	n.s
Maternal height (cm)	162.33 ± 5.36	161.35 ± 5.26	0.74	n.s
BMI (kg/m^2^)	22.61 ± 2.82	20.73 ± 1.92	0.021	*
Gestational weeks (week)	38.71 ± 1.06	39.15 ± 1.5	0.15	n.s
1 h post OGTT (mmol/L)	9.17 ± 2.11	7.33 ± 1.09	7.6e-3	**
2 h post OGTT (mmol/L)	7.54 ± 1.26	6.21 ± 1.07	3.9e-3	**
Fasting cholesterol (mmol/L)	5.83 ± 0.98	5.68 ± 1.2	0.62	n.s
Fasting HDL-c (mmol/L)	2.01 ± 0.44	1.93 ± 0.37	0.60	n.s
Fasting LDL-c (mmol/L)	2.76 ± 0.66	2.78 ± 0.91	0.94	n.s
24-week glycemia (mmol/L)	7.54 ± 1.26	6.21 ± 1.07	3.96e-3	**
Fetal birth weight (g)	3371.9 ± 507.8	3269 ± 433.89	0.566	n.s
Fetal gender (male/female)	11/10	12/8	/	/

Difference analysis was conducted by Wilcoxon rank-sum test, * *P* value < 0.05, ** *P* value < 0.01, n.s. *P* value >  = 0.05

*BMI* Body mass index, *OGTT* Oral glucose tolerance test, *HDL-c* High-density lipoprotein cholesterol, *LDL-c* Low-density lipoprotein cholesterol

### Placenta shows genome-wide methylation alterations associated with type II diabetes mellitus in GDM patients

**Fig. 1:**
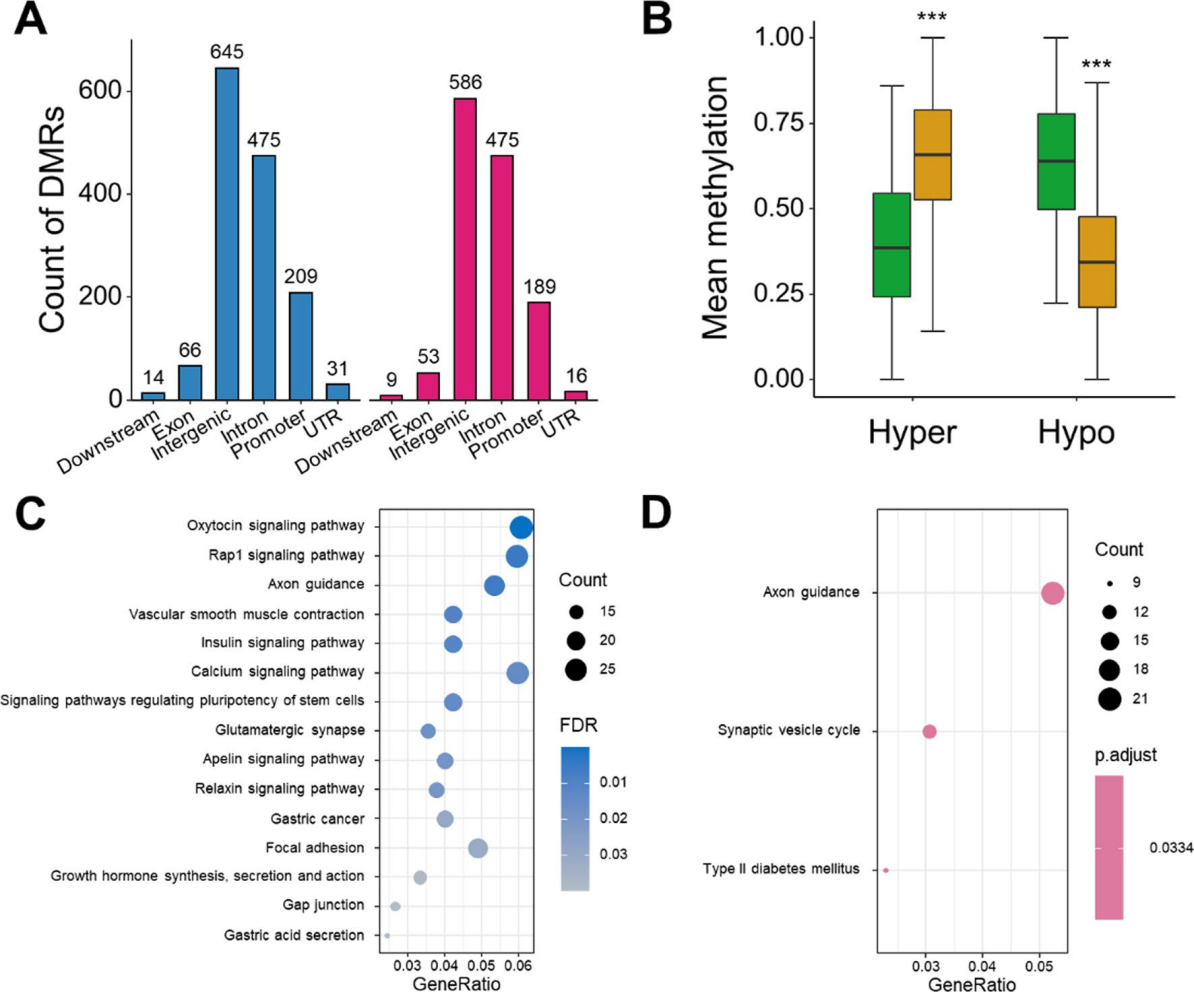
Placenta shows genome-wide methylation alteration associated with glucose metabolism in GDM patients. **A** Genomic annotation of DMRs in placenta. Blue bar represents hyper-DMRs and red bar represents hypo-DMRs. **B** Differences of DMRs mean methylation levels between GDM and control group. Yellow box represents GDM samples and green box represents control samples. *** *P* value < 0.001, Wilcoxon rank-sum test. **C** KEGG pathway enrichment analysis of hyper-DMGs in placenta. **D** KEGG pathway enrichment analysis of hypo-DMGs in placenta

DMRs are genomic regions with differential methylation status across multiple CpG sites, which are considered to play an important role in gene imprinting and regulating the expression of nearby genes that were called differentially methylated genes (DMGs). In placenta samples, after excluding the effect of maternal BMI, a total of 2779 DMRs were identified, including 1446 hyper-methylated DMRs (hyper-DMRs) associated with 1293 hyper-methylated DMGs (hyper-DMGs) and 1333 hypo-methylated DMRs (hypo-DMRs) associated with 1197 hypo-methylated DMGs (hypo-DMGs) (Fig. 1A, Additional file 2: Table S2). Among them, 209 hyper-DMRs and 189 hypo-DMRs were located in promoter regions, respectively. The mean methylation of hyper-DMR and hypo-DMR in GDM and control groups is shown (Fig. 1B). As expected, compared with the control group, the distribution of mean methylation levels of all hyper-DMRs or hypo-DMRs in GDM group showed significant difference (Wilcoxon rank-sum test, *P* value < 0.05). Additionally, hyper-DMRs and hypo-DMRs widely spread across different chromosomes (Additional file 1: Fig. S1C), indicating that the placenta has extensive and significant methylation changes in a genome-wide manner.

To further characterize DMRs identified, we performed functional enrichment analysis using Gene Ontology (GO) terms and identified 401 and 288 significantly enriched terms for hyper-DMGs and hypo-DMGs (FDR < 0.05), respectively (Additional file 2: Table S3, S5). For hyper-DMGs, the enriched pathways mainly included neural and developmental pathways, endocrine-, and immune-related pathways (Additional file 1: Fig. S1D). For hypo-DMGs, the enriched GO terms were similar to that in hyper-DMRs, but also included other ones, such as “cognition,” “long-term memory,” and epigenetic modification-related pathways (Additional file 1: Fig. S1E). Meanwhile, enrichment analysis with Kyoto Encyclopedia of Genes and Genomes (KEGG) database identified 31 and 3 pathways for hyper-DMGs and hypo-DMGs, respectively (Fig. 1C, D, Additional file 2: Table S4, S6, FDR < 0.05). Of the pathways associated with hyper-DMGs, “oxytocin signaling” (29 genes, FDR = 7.14e-05), “calcium signaling” (28 genes, FDR = 5.47e-06), and “insulin signaling” (19 genes, FDR = 8.63e-03) ranked in the top of the list (Fig. 1C). Interestingly, the “type II diabetes mellitus” pathway was significantly enriched in hypo-DMGs (FDR = 0.033), suggesting that GDM may have the epigenetic characteristics of type II diabetes mellitus (Fig. 1D).

### Methylation contributes to expression change of genes associated with insulin signaling pathway

**Fig. 2:**
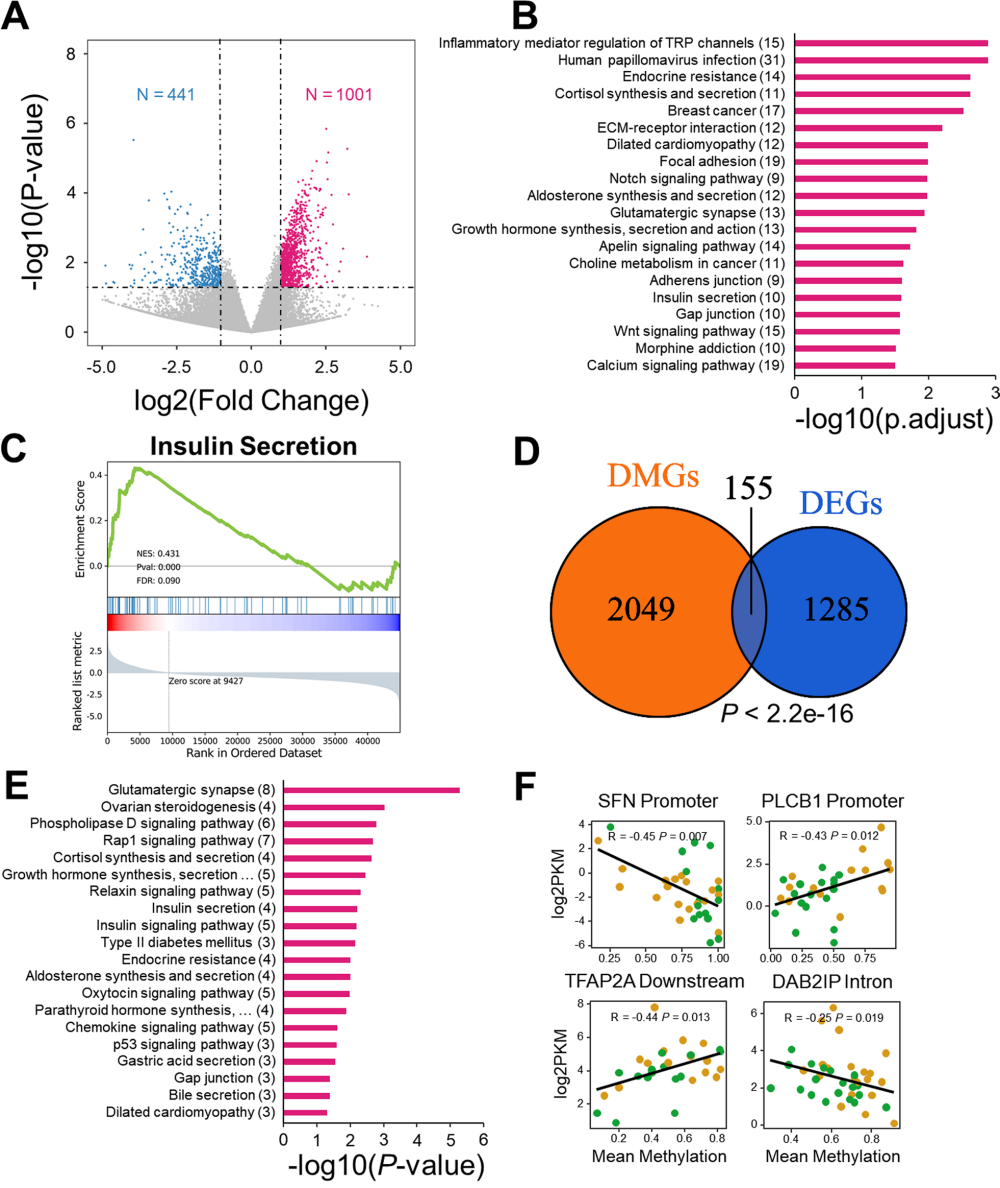
Methylation contributes to expression change of genes associated with insulin signaling pathway. **A** Volcano plot of placenta expression profile. **B** KEGG pathway enrichment analysis of up-regulated genes in placenta. The numbers in the brackets of the ordinate label represent the number of genes involved. **C** GSEA analysis of Insulin secretion pathway in placenta expression profile. **D** The Venn Diagram between DMGs and DEGs in placenta, Fisher’s Exact Test. **E** KEGG pathway enrichment analysis result of shared genes between DMGs and DEGs in placenta, the numbers in the brackets of the ordinate label represent the number of genes involved. **F** Correlation between gene methylation and gene expression in placenta, title of each panel shows the gene name and genomic location. Significance was tested by Spearman’s rank correlation test

To further explore the pathogenesis of GDM, we next performed RNA-seq analysis using placenta samples from the GDM and control groups. After adjustments for multiple testing, we obtain 113 up-regulated and 9 down-regulated genes (FDR < 0.05). Given this number of DEGs is so small, it is challenging to interpret these genes in terms of biological processes and signaling pathways. However, this is not totally uncommon and has been observed in several previous type II diabetes mellitus-related studies [26, 27]. For this reason, researchers have developed the Gene Set Enrichment Analysis (GSEA) method, which was designed to detect modest but coordinate changes in the expression of groups of functionally related genes [28]. This also suggests that top-ranking genes are still good candidates for subsequent analysis, even though individual one of which is not statistically significant. For this reason, we used a *P* value-based threshold (|Fold changes|> 2, *P* value < 0.05) to select DEGs. With this threshold, 1001 genes were up-regulated, and 441 genes were down-regulated (Fig. 2A, Additional file 2: Table S7, S8). The up-regulated genes were annotated to multiple endocrine and metabolic-related pathways, including “endocrine resistance,” “cortisol synthesis, and secretion,” “dilated cardiomyopathy,” and “insulin secretion” (Fig. 2B, Additional file 2: Table S9 and S10). For down-regulated genes, 341 and 32 terms were enriched in GO and KEGG functional enrichment analysis, respectively (Additional file 2: Table S11 and S12). Examples of enriched KEGG terms include “antigen processing and presentation” (FDR = 8.89e-12), and “type I diabetes mellitus” (FDR = 2.81e-10) (Additional file 1: Fig. S2A). GO enrichment analysis showed that pathways related to immune regulation, cell proliferation, and glycolipid metabolism were most enriched (Additional file 1: Fig. S2B).

We next conducted GSEA using expression profile of placenta to detect modest but coordinate changes in the expression of groups of functionally related genes. Of note, 28/40 and 31/32 of KEGG positive and negative enriched pathways were validated by GSEA results, respectively (FDR < 0.25), which show that DGE-based results are largely consistent with that from GSEA in the pathway-level (Additional file 2: Table S13, S14). Interestingly, we discovered two significant insulin-related pathways, “insulin secretion” (FDR = 0.0898, Fig. 2C) and “insulin resistance” (FDR = 0.111, Additional file 1: Fig. S2D). Other positively enriched pathways include the “notch signaling pathway,” “autophagy,” “growth hormone synthesis, secretion and action,” “prolactin signaling pathway,” and “aldosterone synthesis and secretion pathways” (Table 2). Most notably, pathway “type I diabetes mellitus” ranked first in the negative enriched pathways (FDR < 0.001, Additional file 1: Fig. S2E). Other negatively enriched pathways, such as “one carbon pool by folate” (FDR = 3.65e-3), “primary bile acid biosynthesis” (FDR = 6.96e-4), and “folate biosynthesis pathway” (FDR = 0.012), are listed in Table 3.

**Table 2:** Positive enriched pathways of GSEA result for placenta transcriptome (top20)

Pathway	Size	NES	*P* value	FDR
Cortisol synthesis and secretion	64	0.50	< 0.001	0.16
Adherens junction	71	0.49	< 0.001	0.097
Notch signaling pathway	59	0.48	< 0.001	0.083
Autophagy	32	0.47	< 0.001	0.085
Growth hormone synthesis, secretion and action	118	0.46	< 0.001	0.087
ERBB signaling pathway	85	0.46	< 0.001	0.075
Snare interactions in vesicular transport	33	0.45	5.52e-3	0.075
Lysine degradation	63	0.43	< 0.001	0.10
Ovarian steroidogenesis	50	0.43	< 0.001	0.093
Insulin secretion	84	0.43	< 0.001	0.089
Alpha-linolenic acid metabolism	24	0.42	0.0175	0.086
GNRH signaling pathway	92	0.41	< 0.001	0.10
Prolactin signaling pathway	69	0.41	< 0.001	0.094
Inositol phosphate metabolism	73	0.41	< 0.001	0.091
Chronic myeloid leukemia	76	0.41	< 0.001	0.087
Aldosterone synthesis and secretion	97	0.41	< 0.001	0.082
Phosphatidylinositol signaling system	96	0.40	< 0.001	0.092
Hedgehog signaling pathway	56	0.40	< 0.001	0.087
Endometrial cancer	58	0.40	< 0.001	0.089
Acute myeloid leukemia	67	0.40	< 0.001	0.086

Size represents the number of genes enriched in the corresponding pathway

*NES* Normalized enrichment score, *FDR* False discovery rate

**Table 3:** Negative enriched pathways of GSEA result for placenta transcriptome (top20)

Pathway	Size	NES	*P* value	FDR
Type I diabetes mellitus	40	− 0.78	< 0.001	< 0.001
Allograft rejection	34	− 0.76	< 0.001	< 0.001
Ribosome	135	− 0.74	< 0.001	< 0.001
Graft-versus-host disease	36	− 0.74	< 0.001	< 0.001
Intestinal immune network for IGA production	44	− 0.72	< 0.001	< 0.001
Autoimmune thyroid disease	41	− 0.70	< 0.001	1.14e-5
Staphylococcus aureus infection	87	− 0.69	< 0.001	9.77e-5
Asthma	28	− 0.69	< 0.001	8.55e-5
Leishmaniasis	74	− 0.66	< 0.001	4.64e-4
Primary bile acid biosynthesis	17	− 0.65	2.63e-3	6.96e-5
Systemic lupus erythematosus	127	− 0.64	< 0.001	1.00e-3
Inflammatory bowel disease	60	− 0.63	< 0.001	1.09e-3
Rheumatoid arthritis	87	− 0.63	< 0.001	1.01e-3
Thiamine metabolism	15	− 0.6	2.67e-3	1.38e-3
Coronavirus disease	220	− 0.62	< 0.001	1.84e-3
Viral myocarditis	57	− 0.61	< 0.001	1.81e-3
Complement and coagulation cascades	83	− 0.60	< 0.001	2.15e-3
Antigen processing and presentation	69	− 0.60	< 0.001	2.11e-3
Pertussis	76	− 0.59	< 0.001	2.99e-3
One carbon pool by folate	20	− 0.58	2.55e-3	3.65e-3

Size represents the number of genes enriched in the corresponding pathway

*NES* Normalized enrichment score

Next, to investigate whether methylation will affect the expression of genes that may attribute to GDM, we combined methylation data and transcriptome data for an integrated analysis. The analysis indicated that 155 genes showed both methylation changes and expression alternations between the GDM and the control group (*P* value < 2.2e-16, odds ratio = 4.69, Fisher’s Exact Test, Fig. 2D). Though not statistically significant after adjustment for multiple comparisons, the top-ranking pathways enriched in the overlapping genes include “Glutamatergic synapse” (*ADCY5/ADCY7/GRIK4/PLA2G4A/PLCB1/SHANK2/SHANK3/SLC1A6*, *P* value = 5.32e-06), “cortisol synthesis and secretion” (*ADCY5/ADCY7/LDLR/PLCB1*, *P* value = 2.29e-03), “insulin secretion” (*ADCY5/ADCY7/PLCB1/RYR2*, *P* value = 6.28e-3), “insulin signaling pathway” (*HK2/PRKCZ/SH2B2/SHC2/SOCS3*, *P* value = 6.34e-3), “type II diabetes” (*HK2/PRKCZ/SOCS3*, *P* value = 7.10e-3) and “bile secretion” (*ADCY5/ADCY7/LDLR*, *P* value = 0.040) (Fig. 2E). We then selected 10 genes of interest and showed their mean methylation difference, expression change, and associated pathways in Table 4. Besides, from the 155 overlapped gene, we screened out four gene, *SFN*, *PLCB1*, *TFAP2A* and *DAB2IP*, whose gene expression level and gene mean methylation level show significant correlation and functional for glycolipid metabolism (Fig. 2F). Meanwhile, using the GSEA method, placenta CpG methylation generally had a negative regulatory effect on gene expression (*P* value < 0.001, NES = -0.255, Additional file 1: Fig. S2F). In summary, we found that some genes involved in glycolipid metabolism, insulin secretion, and insulin resistance had been epigenetically modified, which might contribute directly or indirectly to GDM.

**Table 4:** Annotation of overlapping genes of placenta DMGs and DEGs

Gene	Delta	log2FC	Pathway
ADCY5	0.5	1.93	Ovarian steroidogenesis
			Cortisol synthesis and secretion
			Insulin secretion
			Bile secretion
B3GALT5	0.12	− 1.64	Glycosphingolipid biosynthesis—globo and isoglobo series
GADD45A	− 0.25	− 1.5	p53 signaling pathway
			Chronic myeloid leukemia
HK2	− 0.67	− 2.92	Insulin signaling pathway
			Type II diabetes mellitus
			Fructose and mannose metabolism
JAG2	0.25	1.81	Endocrine resistance
LDLR	− 0.63	1.31	Cortisol synthesis and secretion
			Aldosterone synthesis and secretion
			Bile secretion
PLA2G4A	0.4	− 1.25	Glutamatergic synapse
			Ovarian steroidogenesis
			Oxytocin signaling pathway
RYR2	− 0.12	1.86	Insulin secretion
			Oxytocin signaling pathway
			Dilated cardiomyopathy]
SH2B2	0.49	1.43	Insulin signaling pathway
SOCS3	− 0.5	− 1.04	Growth hormone synthesis, secretion and action
			Insulin signaling pathway
			Type II diabetes mellitus

*delta* DNA methylation difference, *log2FC* log2(Fold Change), *Pathway* KEGG pathway annotation

### Alterations for umbilical cord blood were related to insulin secretion and resistance

**Fig. 3:**
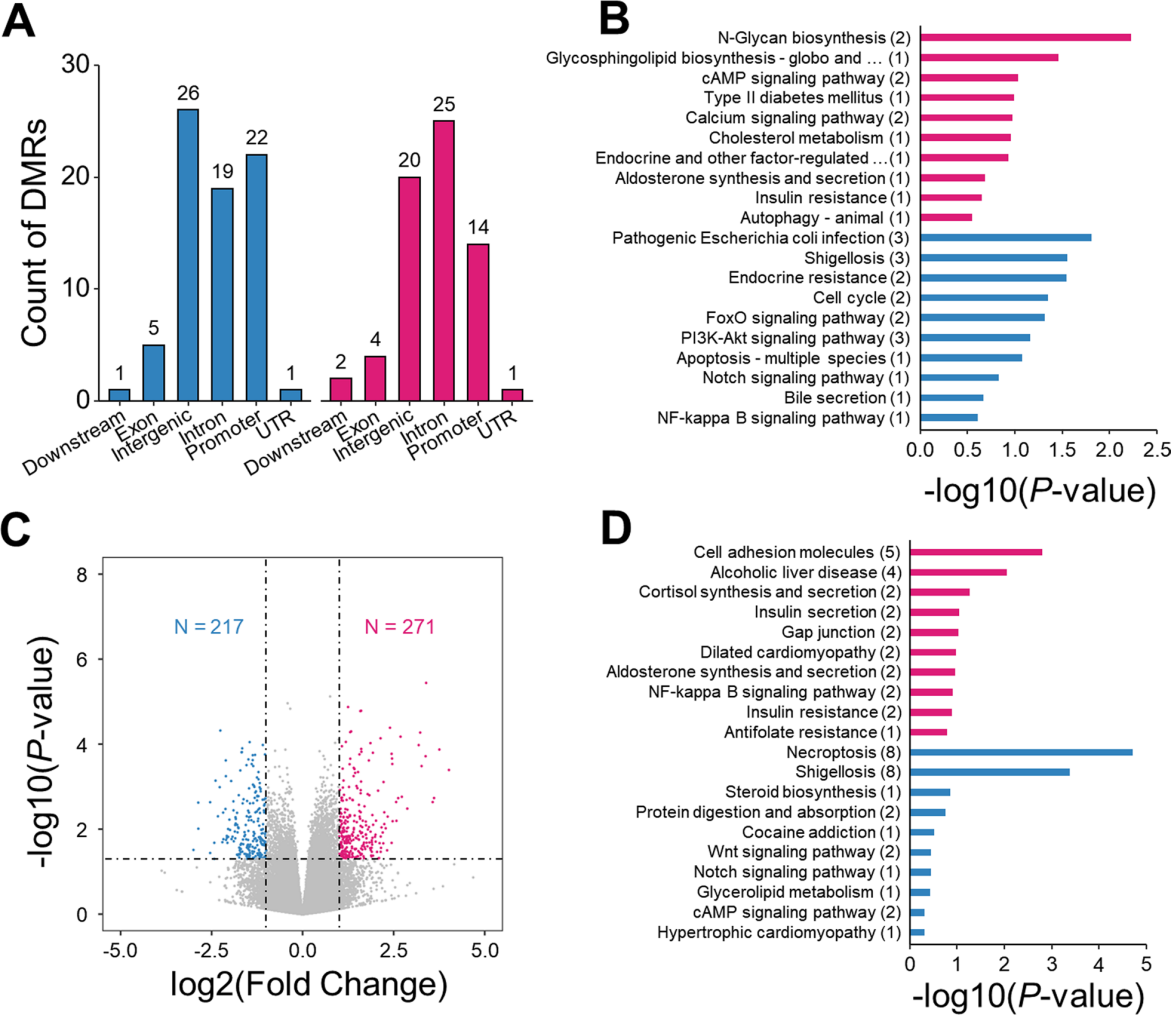
Alterations for umbilical cord blood were related to insulin secretion and resistance. **A** Genomic annotation of DMRs in umbilical cord blood. Blue bar represents hyper-DMRs and red bar represents hypo-DMRs. **B** KEGG pathway enrichment analysis of hypo-DMGs (red bar) and hyper-DMGs (blue bar) of umbilical cord blood. **C** Volcano plot of umbilical cord blood expression profile. **D** KEGG pathway enrichment analysis of up-DEGs (red bar) and hyper-DEGs (blue bar) in umbilical cord blood

To explore whether the exposure to increased glucose level could cause epigenetic changes in fetus, we next conducted RRBS and RNA-seq analyses using the umbilical cord blood paired with placenta described above. Due to the existence of placental barrier, the changes of fetal epigenome were subtler comparing with that in placenta. Genome-wide methylation profiling by RRBS identified far less numbers of DMRs in contrast to that from the paired placenta. Specifically, after excluding the effect of maternal BMI, we detected 75 hyper-DMRs and 66 hypo-DMRs which annotated to 74 hyper-DMGs and 65 hypo-DMGs, respectively (Fig. 3A, B; Additional file 1: Fig. S3A). Next, KEGG pathway enrichment analysis was conducted to identify pathways of differentially methylated genes. Though not statistically significant after adjustment for multiple comparisons, some enlightening pathways were identified in hypo-DMRs (Fig. 3B, red bar), including “cAMP signaling pathway” (*ATP2B3*/*DRD5*), “type II diabetes mellitus” (*PRKCD*), “cholesterol metabolism” (*OSBPL5*), “aldosterone synthesis and secretion” (*ATP2B3*), “insulin resistance” (*PRKCD*). On the other hand, pathways “endocrine resistance” (*JAG2*/*RB1*), “PI3K-Akt signaling pathway” (*BCL2L11*/*LPAR2*/*TCL1A*), and “bile secretion” (*SLC10A2*) were found in hyper-methylated genes (Fig. 3B, blue bar).

RNA-seq analyses of umbilical cord blood samples revealed 271 up-regulated genes and 217 down-regulated genes (|Fold change|> 2, *P* value < 0.05, Additional file 2: Table S15, S16) by comparing GDM patients to the controls (Fig. 3C). KEGG enrichment analysis showed that the up- and down-regulated genes were associated with 21 and 6 pathways, respectively (*P* value < 0.05, Fig. 3D). Interestingly, two pathways, “human papillomavirus infection” and “ovarian steroidogenesis” were overlapped for up-regulated genes between placenta and umbilical cord blood, showing that there is a certain relationship of expression profiles between pregnant women and offspring.

Due to minor changes in gene expression levels, GSEA was then performed as a cutoff-free method for pathway enrichment analysis (Additional file 2: Table S17, S18). In details, “collecting duct acid secretion” (FDR = 0.235), “FC gamma *R*-mediated phagocytosis” (FDR = 0.244), and “staphylococcus aureus infection” (FDR = 0.249) were significantly activated. By contrast, “systemic lupus erythematosus” (FDR = 0.0816), “one carbon pool by folate” (FDR = 0.0844), “fatty acid biosynthesis” (FDR = 0.203), and “primary bile acid biosynthesis” (FDR = 0.239) were significantly repressed. It is worth noting that pathway “one carbon pool by folate” were also enriched in the placenta samples. These results preliminarily show that there is a strong correlation between the current genomic changes and the offspring, and these changes are related to glucose metabolism and insulin, suggesting the possibility of metabolic diseases in the offspring.

In umbilical cord blood, we found one overlapping gene *ZNF423* (DNA methylation delta = − 0.4, log_2_(Fold Change) = − 2.15), whose gene methylation and expression were both altered. Expression of *ZNF423* has been reported directly to be proportional to the size of adipocytes [29]. The gene product of *ZNF423* promotes the transformation of non-adipocytes into adipocytes and inhibits subcutaneous adipogenesis, leading to adipocyte hypertrophy and inflammation, and finally developing to obesity and insulin resistance [30]. The significant down-regulation of *ZNF423* in umbilical cord blood may be related to the higher possibility of suffering obesity in offspring exposed to GDM.

### Significant correlation between the changes of placenta and umbilical cord blood

**Fig. 4:**
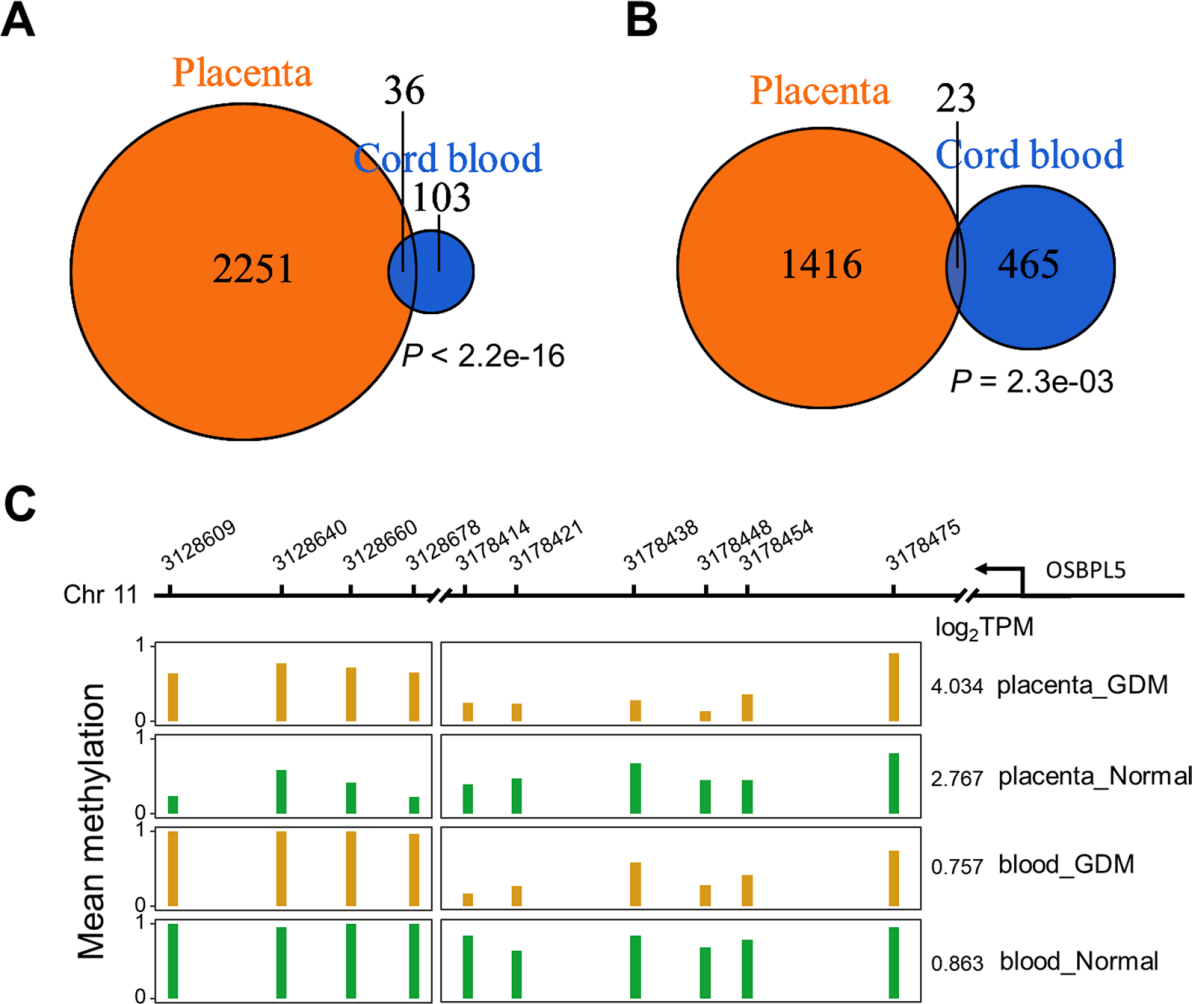
Significant correlation between the changes of placenta and umbilical cord blood, and involves glycolipid metabolism. **A** The Venn Diagram of DMGs in placenta and umbilical cord blood. Significance was tested by Fisher’s Exact Test. **B** The Venn Diagram of DEGs in placenta and umbilical cord blood. Significance was tested by Fisher’s Exact Test. **C** Mean methylation level of OSBPL5 DMR for placenta and umbilical cord blood in different group, log_2_TPM of each group is annotated on the right of each panel. Yellow bar represents GDM samples and green bar represents control samples

After discovering altered pathways shared by umbilical cord blood and placenta samples, we further explored the correlation between placenta and umbilical cord blood through an integrated analysis. The analysis revealed that 36 DMRs co-existed in the placentas and paired umbilical cord bloods (*P* value < 2.2e-16, odds ratio = 9.04, Fisher’s exact test, Fig. 4A). The top-ranking pathways enriched in the shared DMRs include “glycosphingolipid biosynthesis-globo and isoglobo series” (*A4GALT*, *P* value = 0.012), “N-glycan biosynthesis” (*ALG10*, *P* value = 0.042) and “cholesterol metabolism” (*OSBPL5*, *P* value = 0.042).

We then investigated altered gene expressions in placentas and paired umbilical cord blood samples by comparing GDM patients to the controls. The results demonstrated that 23 genes were overlapped between placenta and paired umbilical cord blood (*P* value = 2.3e-03, odds ratio = 2.04, Fisher’s exact test, Fig. 4B). The top-ranking pathways enriched in the shared genes include “ECM-receptor interaction” (*FRAS1*/*SPP1*, *P* value = 4.92e-03), “growth hormone synthesis, secretion and action” (*ADCY5*/*SOCS3*, *P* value = 8.84e-03) and “type II diabetes mellitus” (*SOCS3*, *P* value = 0.055).

The gene *OSBPL5* encode a protein which belongs to the oxysterol-binding protein family, and proteins in this family are critical to maintenance of cholesterol balance in human body [31]. Comparison of the methylation levels of *OSBPL5* across multiple specimens is illustrated in Fig. 4C. The DMR in placenta was annotated into one of the exons in the *OSBPL5* gene, including four CpG sites (DNA methylation delta = 0.35; Fig. 4C left panel). Likewise, the DMR in umbilical cord blood located in an intron, including six CpG sites (DNA methylation delta = − 0.38; Fig. 4C right panel). Profiling of transcriptome showed that expression of *OSBPL5* (log_2_TPM) in the placenta of GDM patient was 2.4-fold of that in the control group (GDM 4.034 vs. control 2.767), but the difference was small in umbilical cord blood (GDM 0.757 vs. control 0.863). These results suggest that the methylation and expression profile of umbilical cord blood are closely related to that in placenta, and the genes in glucose metabolism-related pathway are more likely to be regulated by DNA methylation.

### Discussion

To the best of our knowledge, this is the first study that use epigenomic and transcriptomic assays to characterize placenta and the paired umbilical cord blood samples from GDM patients and controls. Our data demonstrated that placenta undergoes extensive methylation changes in genomic regions that are related to glucose metabolism-related pathways. Remarkably, methylation-altered genes in umbilical cord blood were associated with pathways insulin resistance and insulin secretion. And we also found that DMGs and DEGs were significantly overlapped between placenta and umbilical cord blood, indicating that the GDM conditions could affect fetus.

An early study found that insulin resistance in GDM patients led to a compensatory increase of the synthesis and secretion of insulin, which can promote the absorption and metabolism of blood glucose in islet β-cells [32]. However, when insulin resistance increases to a certain level, over-secreted insulin fails to maintain blood glucose levels in a normal range [33] and GDM patients could display hyperglycemia and hyperinsulinemia simultaneously [34]. GSEA result of placental RNA-seq data showed that several genes associated with the insulin secretion and the insulin resistance pathways were significantly up-regulated. More importantly, genes with both DNA methylation alternations and expression changes were identified, including *PRKCZ, ADCY5, CACNA1C, PLCB1*. The DNA methylation alternations in those genes might contribute to expression changes of these gene, which further aggravates insulin resistance and insulin secretion.

At the beginning of pregnancy, the placenta releases hormones into the blood of pregnant women, raising the blood glucose concentration to ensure adequate nutrition for the fetus [35, 36]. Our data showed that many genes related with hormone production and secretion were highly expressed in placenta of GDM patients, which involved in “cortisol synthesis and secretion,” “growth hormone synthesis and secretion” and “aldosterone synthesis and secretion pathway” (Table 2). However, it is reported that excessive cortisol levels in pregnant women’s blood can promote anxiety [37], increase the risk of obesity in offspring [38] and reduce children’s cognitive ability [39]. Our investigation discovered decreased methylation levels in multiple genes of the cortisol synthesis and secretion pathway, suggesting that cortisol levels could be altered in GDM patients through placenta. This hypothesis is further supported by the GSEA result of RNA-seq data, which listed pathway “cortisol synthesis and secret” as one of the most positively enriched pathways (Additional file 1: Fig. S2C). The genes of which both methylation statuses and expression levels varied in this pathway included *CACNA1C*, *LDLR*, and *ADCY9*. The *CACNA1C* gene encodes an alpha-1 subunit of a voltage-dependent calcium channel and is related to the exocytosis of many hormones. Xu et al. [40] reported that miR-153 worked as a negative regulator for the expression of *CACNA1C*, which further increased the secretion of insulin. An independent GWAS study revealed that *CACNA1C* gene is associated with diabetic cataract [41]. Besides, the co-binding of low-density lipoprotein receptor (LDLR) and insulin receptor (IR) reduces the ability of LDLR to clean up LDLR in blood, and insulin can destroy this co-binding [42].

Moreover, compared with control pregnancy, GDM patients had significantly lower serum total bile acids at 24 weeks of gestation (Additional file 1: Fig. S4A) and at 40 weeks of gestation (Additional file 1: Fig. S4B) (Wilcoxon rank-sum test, *P* value < 0.05). Previous studies have shown that bile acids can activate glucose induced insulin secretion [43] and have a strong correlation with insulin resistance and nonalcoholic fatty liver [44]. Based on the technique of liquid chromatography, Li et al. reported that lower levels of serum bile acids in early pregnancy were independently associated with an increased risk of GDM in Chinese pregnant women [45]. At the same time, our GSEA analysis also showed that several genes associated with the primary bile acid biosynthesis pathway were significantly down-regulated in placental tissues (Additional file 1: Fig. S4C). Expression of *CH25H*, *CYP7B1* and *HSD3B7* were significantly down-regulated in core enrichment genes, which played an important role in catalytic synthesis of bile acids. *CYP7B1* is also involved in the synthesis of dehydroepiandrosterone and pregnenolone [46]. Both dehydroepiandrosterone and pregnenolone are related to hippocampus-associated memory and learning, which may reveal the relationship between GDM patients and the risk of memory decline. We believe that this is the first report to connect bile acid metabolism with GDM using placental high-throughput sequencing data. Notably, bile acid has been reported for the treatment of patients with obesity and diabetes [47], suggesting that bile acids can potentially be used in pregnant women to prevent and treat GDM in the future.

In placental tissues, 155 genes were differentially methylated and differentially expressed (partially displayed in Table 4), and some of these genes were involved in key metabolic pathway. From those genes, we manifested that the expression of three critical genes in the type II diabetes pathway was altered, including *HK2* (down-regulated), *PRKCZ* (up-regulated) and *SOCS3* (down-regulated). *HK2* is the key gene of glycolysis, and its down-regulation has been shown directly leading to the reduction of glucose metabolism [48]. *SOCS3* can inhibit insulin secretion, and down-regulation of this gene promote insulin secretion, resulting in insulin resistance [49]. The activation of *PRKCZ* depends on insulin, and over-expression of this gene affects the expression of the insulin-like growth factor 1 receptor (*IGF1R*) gene [50]. Additionally, previous research show that genes illustrated in Fig. 2F play key roles in insulin secretion, lipid droplet formation and are associated with type II diabetes [51–53]. Taken together, those results suggested that the pathogenesis of GDM may be similar to type II diabetes, characterized with excessive insulin secretion and insulin resistance, and DNA methylation plays an important role in the process.

Previous studies have indicated that the methylation levels of metabolism-related genes or genes involved in pathways could be changed after exposing to persistent high-glucose levels in pregnant women with GDM, which is termed “metabolic programming” [54–56]. In present results, several pathways showed descending methylation levels in placenta of GDM patients, including the “aldosterone synthesis and secretion pathway,” “insulin resistance” and “type II diabetes mellitus pathway”*.* Besides, for up-regulated genes in placenta of GDM patients, enriched pathways included “EGFR tyrosine kinase inhibitor resistance,” “insulin secretion and “growth hormone synthesis, secretion and action”*.* Our results indicated that the glucose and lipid metabolism of offspring were probably affected by intrauterine exposure to GDM, both in epigenetic and expression levels. Furthermore, there was a significant relevance between the changes of placenta and umbilical cord blood, regardless differentially methylated genes (Fig. 4A) or differentially expressed genes (Fig. 4B). Most notably, genes in the one carbon pool by folate pathway were significantly down-regulated in placentas and umbilical cord bloods, indicating that both pregnant women and fetuses with GDM may have the characteristics of folate deficiency. However, a meta-analysis regarding to the correlation between folic acid and the pathogenesis of GDM provides controversial results [57]. Larger cohort studies are necessary to explore the correlations. The above results show that there are apparent associations between the methylome and the transcriptome in placenta and umbilical cord blood, which share the common characteristics of insulin resistance and type II diabetes.

Still, there are few limitations in this study. Firstly, due to the complexity of placenta dissection structure, it is inevitable to obtain a variety of tissues and cell types during sampling. Using single-cell sequencing technology or spatial transcriptome technology will make a more in-depth study on the pathogenesis and mother–child interaction of GDM comparing with traditional bulk RNA-seq. In addition, in differentially gene expression analysis, we did not correct *P* value by FDR method, because very few genes (placenta 122, umbilical cord blood 0) had significant differences with FDR cutoff of 5%. Therefore, we used GSEA method to support our point of view, which results are largely consistent with DGE-based results. Finally, as confounding factor, BMI weights much in present research, and we did not find a suitable tool to exclude the influence of BMI during calling DMRs. Instead, we divided samples into two groups with equal number of samples, BMI-high and BMI-low, and used DSS to identify BMI-related DMRs, which were removed from subsequent analysis.

## Conclusions

In summary, our study systematically profiled the methylome and transcriptome of placenta and umbilical cord blood of pregnant women with or without GDM, and investigated the relationship between methylation and gene expression. Our results provide detailed molecular changes among GDM patients compared with the controls, which pave the way for the further investigation of GDM pathogenicity.

## Materials and methods

### Patient recruitment

Pregnant women were recruited at Hangzhou Women’s Hospital (Hangzhou Maternity and Child Health Care Hospital, China) from January 1, 2020 to May 31, 2020. All participants signed a written consent forms and this study was approved by the ethics committee of Hangzhou Women’s Hospital. GDM is diagnosed according to the International Association of Diabetes and Pregnancy Study Groups (IADPSG) guidelines. Specifically, at least one of the following conditions must be met: 1. Fasting blood glucose ≥ 5.1 mmol/L; 2. Blood glucose ≥ 10.0 mmol/L one hour after the oral glucose tolerance test (OGTT); 3. Blood glucose ≥ 8.5 mmol/L two hours post the OGTT [58]. Pregnant women who met the following conditions were enrolled in this study: (1) age 24–39; (2) singleton pregnancy; (3) full-term birth (37–40 weeks of gestation); (4) detailed information for the OGTT; (5) records showing that the perinatal examination, delivery and infant physical examination were conducted in our hospital. Pregnant women with normal blood glucose and no history of adverse diseases were recruited in the control group. We excluded the pregnant women with the following conditions from our study: (1) having chronic diabetes complicated with pregnancy or preeclampsia. (2) suffering from liver or kidney dysfunctions, or other chronic diseases which required long-term drug treatment; (3) showing a mental disorder or a serious infection; (4) smoking, drinking and drug abuse during pregnancy; (5) fetal chromosome abnormalities; (6) lacking registration information in the Women’s and Children’s Insurance Systems. The difference of physiological parameters of pregnant women and newborns between GDM group and control group was performed by Wilcoxon rank-sum test.

### Placenta and umbilical cord blood sampling

Within 15 min after delivery, 5 ml of neonatal umbilical cord blood was collected using two PAXGenRNA sampling tubes (2.5 ml/tube), and collected samples were stored at – 80 ℃ [59]. The placental tissues were collected from both the maternal and fetal side. Specifically, using a clean scalpel, one full-thick placental tissue was incised 5 cm from the perimeter of the placenta (a size of about 1 × 1 × 2 cm, including maternal and fetal side). Then, the tissues were rinsed with phosphate buffer (PBS) or normal saline until no visible blood and stored at – 80 ℃ in a cryopreservation tube [60]. DNA or RNA was extracted after homogenizing the tissues together.

### RRBS and RNA-seq libraries preparation and sequencing

Genomic DNA of placenta and umbilical cord blood specimens was extracted by using the TIANamp genomic DNA kit (Cat. No. DP304-02, TIANGEN Biotech CO. LTD., Beijing, China) per the manufacture’s recommendations. 50–300 ng of purified genomic DNA was utilized to generate RRBS libraries as previously described [61]. Indexed RRBS libraries were pooled accordingly and sequenced in a NovaSeq 6000 sequencer (Illumina, USA) with 100-base paired-end reads. Similarly, total RNAs were isolated using a miRNeasy Mini kit (QIAGEN, Germany) according to the manufacture’s recommendations. We used the Ribo-off rRNA depletion kit (Cat. No. N406-01, Vazyme Biotech Co. Ltd, Nanjing, China) to remove ribosomal RNA from total RNAs following the manufacturer’s instructions. To generate RNA-seq libraries, 10–100 ng rRNA-depleted RNAs were utilized by using the VAHTS total RNA-seq (H/M/R) library prep kit for Illumina (Cat. No. NR603-01, Vazyme Biotech Co. Ltd, Nanjing, China) per the manufacturer’s instructions. We sequenced the RNA-seq libraries in the NovaSeq 6000 sequencer with 100-base paired-end reads.

The sequencing data volume of a single sample is around 8 gigabases (Gb) and the average sequencing depth of the target region of the enzyme digestion fragment is > 30 × , the coverage proportion of CpG region is > 40%, and the total data volume is 100 Gb. Similarly, transcriptome profile for each placenta and umbilical cord blood sample was also conducted. VAHTSTM Total RNA-seq (H/M/R) Library Prep Kit for Illumina® based on Ribo-zero method was used to constructed transcriptome RNA library and high-throughput sequencing was performed subsequently. 10 Gb volume of transcriptome data for each sample was necessary.

### Data processing

For RRBS data, FastQC (version 0.11.9) [62] software was used to evaluate the quality of the original RRBS sequencing reads, and TrimGalore (version 0.6.5) [63] was further applied to trim adapters and low-quality bases. The trimmed reads were then aligned to the hg19 genome using BSMAP [64] software. Methylation calling of BAM file obtained in the previous step was conducted by MethylDackel [65]. R package DSS [66] was used to discover de novo DMRs. In this research, DMRs were defined as regions whose mean methylation level across CpG sites increased or decreased > 0.1, width > 50 bp, CpG number > 3, and *P* value < 0.05. Specifically, statistical tests for differential methylation at each CpG site were performed by function “DMLtest” with default parameters, which output was then processed using function “callDMR” (parameters: delta = 0.1, p.threshold = 0.05) to call DMRs-GDM by comparing GDM group with control group. Meanwhile, to eliminate the influence of confounding factors, we divided samples into two groups with equal number of samples, BMI-high and BMI-low, and used DSS to identify BMI-related DMRs, which were removed from subsequent analysis. Finally, DMRs were annotated using R package ChIPseeker [67] with default parameters to find differentially methylated genes (DMGs) and genomic distribution of DMRs.

As for RNA-seq data, quality control and base trimming were similarly conducted by using FastQC and TrimGalore, respectively. STAR (2.6.1d) [68] was used to map the quality-controlled reads to the hg19 genome. Then, the transcripts were quantified by using software kallisto [69], and the expression abundance of transcripts was generated accordingly. RNASeQC is used to count the quality of genome alignment. Subsequently, using the output of kallisto, gene expression levels were estimated from the abundance of transcription level by function “tximport” of *R* package tximport [70]. Gene differential expression analysis between GDM group and control group was performed by function DESeq of *R* package DESeq2 [71] using default parameters. Briefly, dispersion was estimated by fitting a dispersion-mean relation via a robust gamma-family GLM (General linear model); library size was estimated by the standard median ratio method introduced in DESeq; finally, the GLM coefficients were tested for significance using Wald test. Meanwhile, BMI was added as a covariate in the difference analysis to exclude the influence of confounding factors.

The unique mapping ratio of RRBS and RNA-seq data were illustrated in Additional file 1: Fig. S1A, B, respectively. All quality control table of RRBS and RNA-seq data were listed in Additional file 2: Table S1.1–S1.4.

### Functional enrichment analysis

The genomic annotation of DMRs was conducted by *R* package ChIPseeker [67] and consequently were grouped to 6 classes: promoter, exon, intron, intergenic, UTR (untranslated region) and downstream regions. To investigate the enriched functional pathways of DMGs and DEGs, functional enrichment analysis of GO (Gene Ontology) biological process and KEGG pathway annotation was performed by the R package clusterProfiler [72] (parameters: pvalueCutoff = 0.05, qvalueCutoff = 0.05, pAdjustMethod = “fdr”).

Gene Set Enrichment Analysis (GSEA) was used to detect modest but coordinate changes in the expression of groups of functionally related genes [73]. Java command line tool gsea (http://www.gsea-msigdb.org/gsea/msigdb/download_file.jsp?filePath=/resources/software/gsea2-2.2.4.jar) was used to find the key pathways in gene expression profiles of placenta and umbilical cord blood (parameters: xtools.gsea.GseaPreranked -scoring_scheme weighted -collapse true -mode Max_probe -norm None -nperm 1000 -include_only_symbols true make_sets true -plot_top_x 20 -rnd_seed timestamp -set_max 3000 -set_min 15 -zip_report false -gui false). Specifically, all genes in gene expression profile were firstly sorted by *Z*-scores, which were converted from *P* values and associated log2 fold change using function *qnorm*. Then, enrichment significance test of each KEGG pathway was performed by GSEA, and *P* values of all pathways were adjusted for multiple testing. According to official advice, pathways with FDR < 0.25 were considered statistically significant.

### Integrating analysis

To explore the effect of DNA methylation on gene expression under GDM environment, we compared differentially methylated genes and differentially expressed genes in a tissue of interest, including placenta and umbilical cord blood. A gene was counted as an overlap if it was differentially expressed and near DMR. The statistical significance of overlapping was tested by Fisher’s exact test with all genes in gene expression profile as a background. Similarly, we compared DMGs in placenta and umbilical cord blood, as well as DEGs in placenta and umbilical cord blood. Spearman’s rank correlation test was used to assess the significance of correlation between mean methylation level of DMGs and mean gene expression level of DEGs across samples. The biological function of overlapped genes was then evaluated by GO/KEGG functional enrichment analysis.

## Supplementary Information

**Additional file1: Fig. S1** Placenta shows genome-wide methylation alteration associated with glucose metabolism in GDM patients. Unique mapping ratio of placenta and blood samples for **A** RRBS data and **B** RNA-seq data. **C** Genomic distribution of DMRs in placenta. GO pathway enrichment result of placenta for **D** hyper-DMGs and **E** hypo-DMGs. The number in brackets represents the number of enriched genes. **Fig. S2** Methylation contributes to expression change of genes associated with insulin signaling pathway. **A** KEGG pathway enrichment analysis of down-DMGs in placenta. **B** “Cortisol synthesis and secretion” **C** “Adherens junction,” **D** “Insulin resistance” and **E** “Type I diabetes mellitus” pathway enrichment result of placenta expression profile GSEA analysis. **F** GSEA analysis of placenta DMGs to placenta expression profile. **Fig. S3** Alterations for umbilical cord blood were related to insulin secretion and resistance **A** Genomic distribution of DMRs in umbilical cord blood. **B** Differences of DMRs mean methylation levels between GDM and control umbilical cord blood samples. Yellow box represents GDM samples and green box represents control samples. *** *P* value < 0.001, Wilcoxon rank-sum test. **Fig. S4** Differential characteristics of primary bile acid synthesis and autophagy between GDM and control samples. Total bile acid concentration between GDM and control sample in **A** 24 gestational week and **B** 40 gestational week. Wilcoxon rank-sum test. **C** ‘primary bile acid biosynthesis’ and **D** ‘Autophagy’ pathway enrichment result of placenta expression profile GSEA analysis.

**Additional file2: Table S1.1** Quality control table of placenta RRBS data. **Table S1.2** Quality control table of umbilical cord blood RRBS data. **Table S1.3** Quality control table of placenta RNA-seq data. **Table S1.4** Quality control table of umbilical cord blood RNA-seq data. **Table S2** The 2779 differential methylation regions of placenta. **Table S3** GO pathway enrichment of hyper-DMGs of placenta. **Table S4** KEGG pathway enrichment of hyper-DMGs of placenta. **Table S5** GO pathway enrichment of hypo-DMGs of placenta. **Table S6** KEGG pathway enrichment of hypo-DMGs of placenta. **Table S7** Up-regulated differentially expressed genes of placenta. **Table S8** Down-regulated differentially expressed genes of placenta. **Table S9** GO pathway enrichment of UP-DEGs of placenta. **Table S10** KEGG pathway enrichment of UP-DEGs of placenta. **Table S11** GO pathway enrichment of DN-DEGs of placenta. **Table S12** KEGG pathway enrichment of DN-DEGs of placenta. **Table S13** Positive enriched pathways of GSEA for placenta gene expression profile. **Table S14** Negative enriched pathways of GSEA for placenta gene expression profile. **Table S15** Up-regulated differentially expressed genes of umbilical cord blood. **Table S16** Down-regulated differentially expressed genes of umbilical cord blood. **Table S17** Positive enriched pathways of GSEA for umbilical cord blood gene expression profile. **Table S18** Negative enriched pathways of GSEA for umbilical cord blood gene expression profile.

## Data Availability

The datasets used and/or analyzed during the current study are available from the corresponding author on reasonable request.

## Abbreviations

GDM: Gestational diabetes mellitus
RRBS: Reduced representation bisulfite sequencing
DMRs: Differentially methylated regions
DEGs: Differentially expressed genes
DMGs: Differentially methylated genes
BMI: Body mass index
MeDIP: Methylated DNA immunoprecipitation
NGS: Next-generation sequencing
OGTT: Oral glucose tolerance test
HDL-c: High-density lipoprotein cholesterol
LDL-c: Low-density lipoprotein cholesterol
hyper-DMRs: Hyper-methylated DMRs
hyper-DMGs: Hyper-methylated DMGs
hypo-DMRs: Hypo-methylated DMRs
hypo-DMGs: Hypo-methylated DMGs
GO: Gene ontology
FDR: False discovery rate
KEGG: Kyoto encyclopedia of genes and genomes
GSEA: Gene set enrichment analysis
NES: Normalized enrichment score
IADPSG: International association of diabetes and pregnancy study groups
PBS: Phosphate buffer
GLM: General linear model
UTR: Untranslated region
