# Supplementary material for: DNA methylation and expression profiles of placenta and umbilical cord blood reveal the characteristics of gestational diabetes mellitus patients and offspring
Source: Clin Epigenetics. 2022 May 23;14:69. doi: 10.1186/s13148-022-01289-5 (PMC9126248; doi:10.1186/s13148-022-01289-5)
Supplement: Supplementary file 1 — Additional file1: Fig. S1 Placenta shows genome-wide methylation alteration associated with glucose metabolism in GDM patients. Unique mapping ratio of placenta and blood samples for A RRBS data and B RNA-seq data. C Genomic distribution of DMRs in placenta. GO pathway enrichment result of placenta for D hyper-DMGs and E hypo-DMGs. The number in brackets represents the number of enriched genes. Fig. S2 Methylation contributes to expression change of genes associated with insulin signaling pathway. A KEGG pathway enrichment analysis of down-DMGs in placenta. B “Cortisol synthesis and secretion” C “Adherens junction,” D “Insulin resistance” and E “Type I diabetes mellitus” pathway enrichment result of placenta expression profile GSEA analysis. F GSEA analysis of placenta DMGs to placenta expression profile. Fig. S3 Alterations for umbilical cord blood were related to insulin secretion and resistance A Genomic distribution of DMRs in umbilical cord blood. B Differences of DMRs mean methylation levels between GDM and control umbilical cord blood samples. Yellow box represents GDM samples and green box represents control samples. *** P value < 0.001, Wilcoxon rank-sum test. Fig. S4 Differential characteristics of primary bile acid synthesis and autophagy between GDM and control samples. Total bile acid concentration between GDM and control sample in A 24 gestational week and B 40 gestational week. Wilcoxon rank-sum test. C ‘primary bile acid biosynthesis’ and D ‘Autophagy’ pathway enrichment result of placenta expression profile GSEA analysis. [file 13148_2022_1289_MOESM1_ESM.pdf]

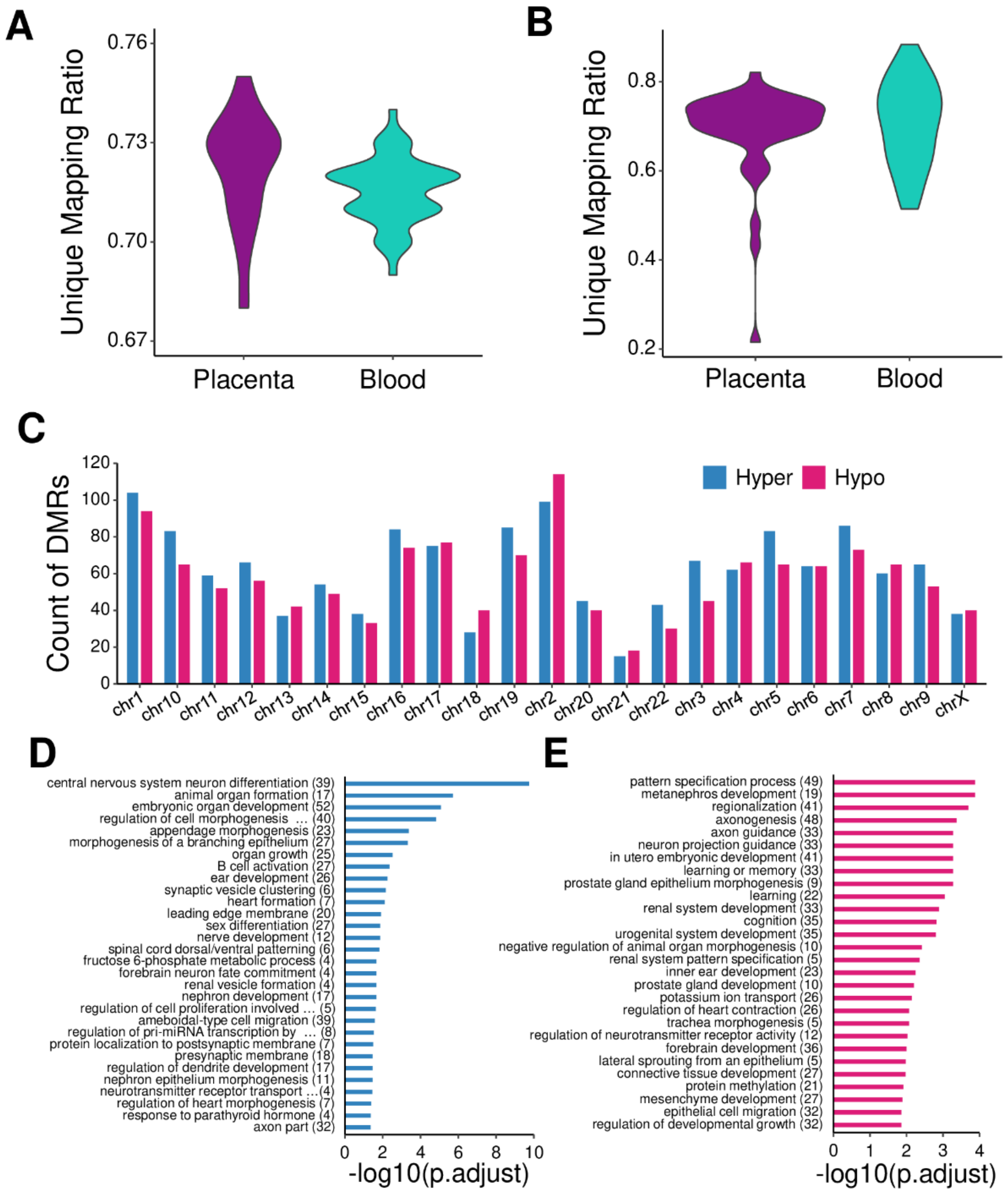

Supplementary Figure 1. Placenta shows genome-wide methylation alteration associated to glucose metabolism in GDM patients. Unique mapping ratio of placenta and blood samples for **(A)** RRBS data and **(B)** RNA-seq data. **(C)** Genomic distribution of DMRs in placenta. GO pathway enrichment result of placenta for **(D)** hyper-DMGs and **(E)** hypo-DMGs. The number in brackets represents the number of enriched genes.

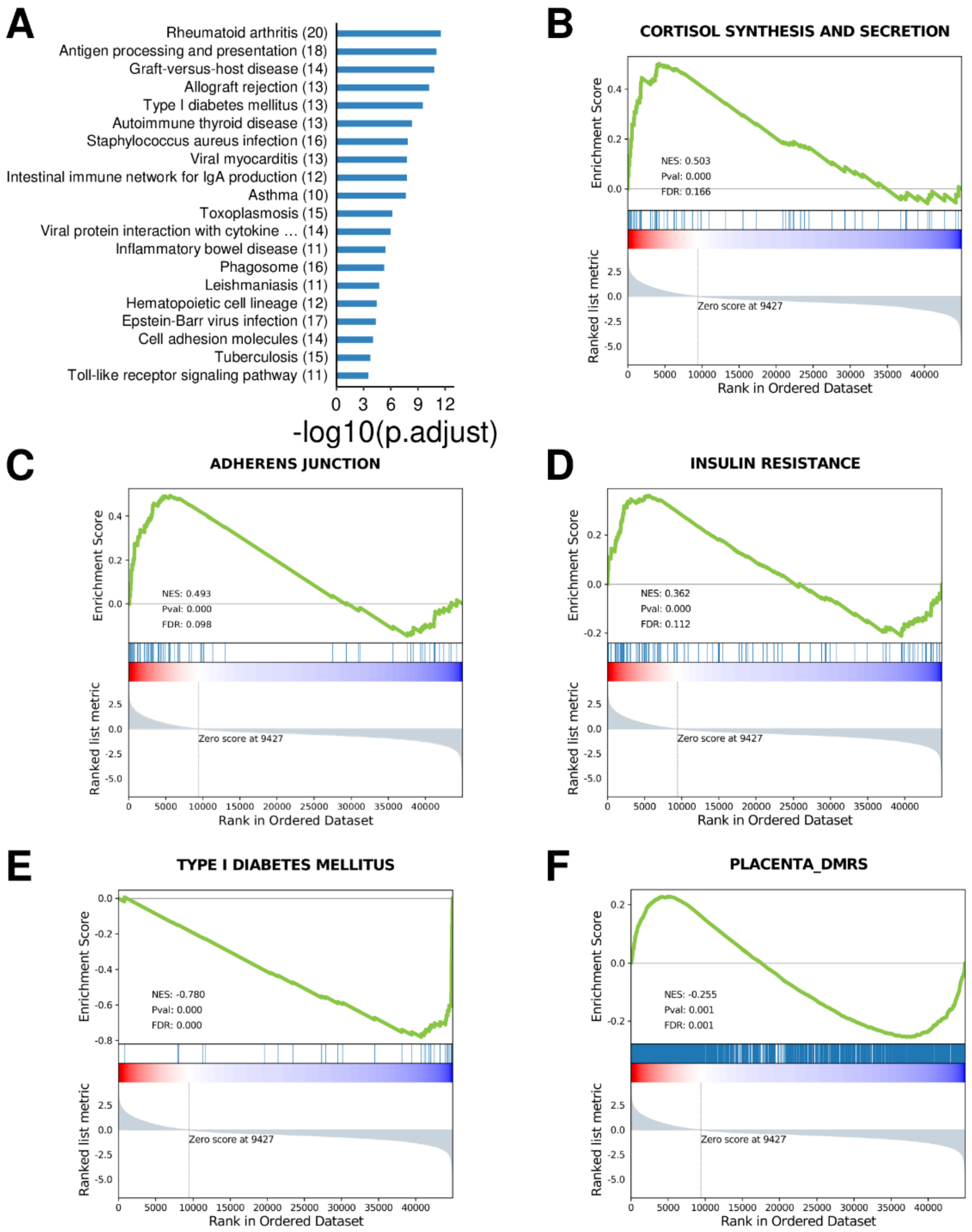

Supplementary Figure 2. Methylation contributes to expression change of genes associated with insulin signaling pathway. (A) KEGG pathway enrichment analysis of down-DMGs in placenta. **(B)** "Cortisol synthesis and secretion" **(C)** "Adherens junction", **(D)** "Insulin resistance" and **(E)** "Type I diabetes mellitus" pathway enrichment result of placenta expression profile GSEA analysis. **(F)** GSEA analysis of placenta DMGs to placenta expression profile.

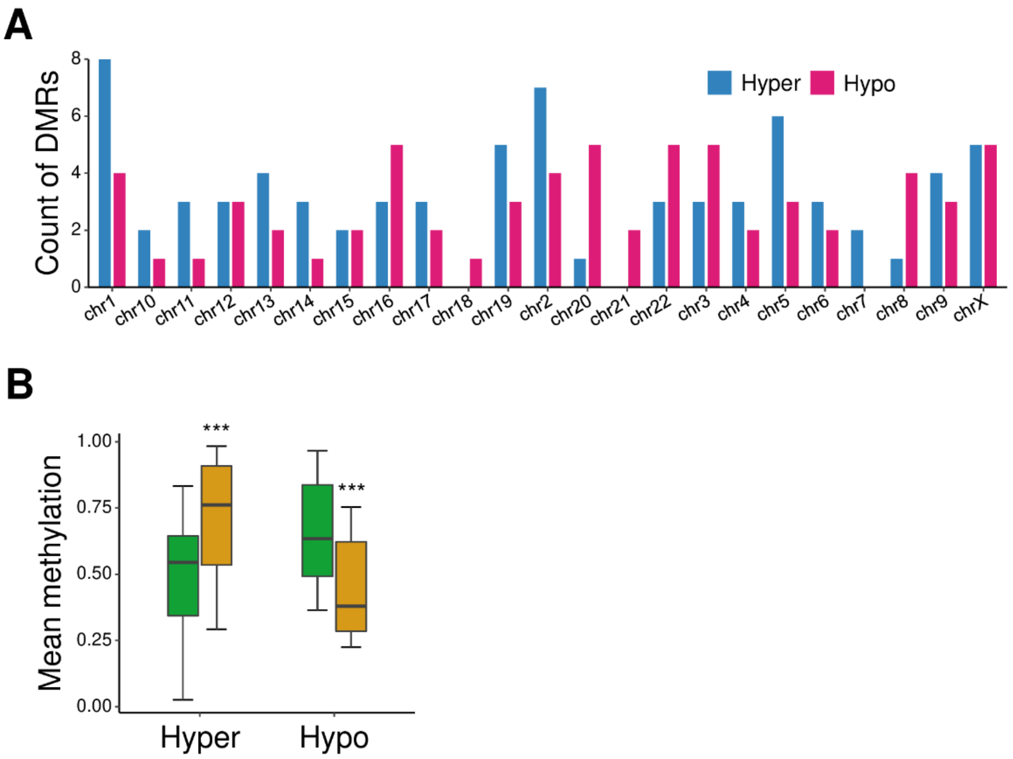

Supplementary Figure 3. Alterations for umbilical cord blood were related to insulin secretion and resistance (A) Genomic distribution of DMRs in umbilical cord blood. **(B)** Differences of DMRs mean methylation levels between GDM and control umbilical cord blood samples. Yellow box represents GDM samples and green box represents control samples. *** P-value < 0.001, Wilcoxon rank-sum test.

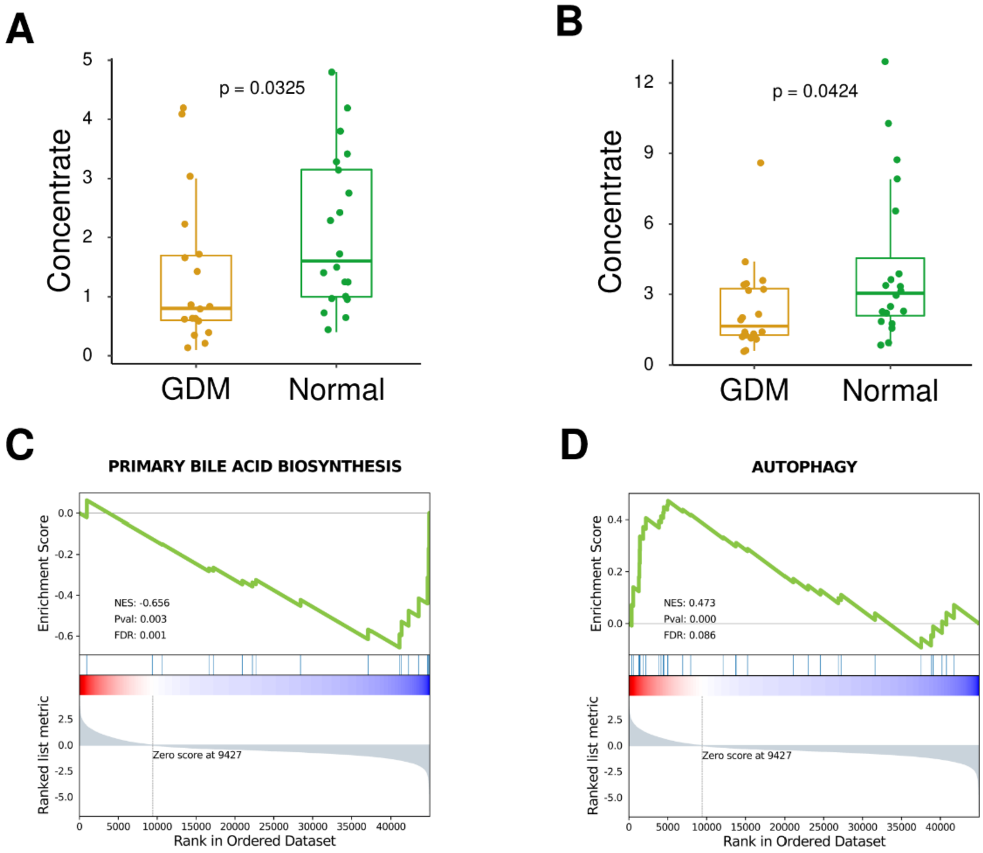

Supplementary Figure 4. Differential characteristics of primary bile acid synthesis and autophagy between GDM and control samples. Total bile acid concentration between GDM and control sample in **(A)** 24 gestational week and **(B)** 40 gestational week. Wilcoxon rank-sum test. **(C)** 'primary bile acid biosynthesis' and **(D)** 'Autophagy' pathway enrichment result of placenta expression profile GSEA analysis.
